# Targeting NOX4 disrupts the resistance of papillary thyroid carcinoma to chemotherapeutic drugs and lenvatinib

**DOI:** 10.1038/s41420-022-00994-7

**Published:** 2022-04-08

**Authors:** Ping Tang, Jianfeng Sheng, Xiujuan Peng, Renfei Zhang, Tao Xu, Jun Hu, Yuexi Kang, Baiyi Wu, Hao Dang

**Affiliations:** 1 Department of Thyroid, Head, Neck and Maxillofacial Surgery, the Third Hospital of Mianyang (Sichuan Mental Health Center), No.190 The East Jiannan Road, Mianyang, 621000 Sichuan China; 2 Department of Clinical Laboratory, the third Hospital of Mianyang (Sichuan Mental Health Center), No.190 The East Jiannan Road, Mianyang, 621000 Sichuan China; 3 College of Basic Medicine, Southwest Medical University, No. 1, Section 1, Xianglin Road, Longmatan District, Luzhou, 646000 Sichuan China

**Keywords:** Thyroid cancer, Cancer therapy

## Abstract

Advanced differentiated thyroid cancer cells are subjected to extreme nutritional starvation which contributes to develop resistance to treatments; however, the underlying mechanism remains unclear. Cells were subjected to serum deprivation by culture in medium containing 0.5% fetal bovine serum. A CCK8 assay, cell death Detection ELISAPLUS kit, and PI staining were conducted to determine cell viability, cell apoptosis, and cell cycle, respectively. NADPH oxidase 4 (NOX4) knockdown–stable cell lines were generated by lentivirus-mediated shRNA knockdown in BCPAP cells and TPC-1 cells. Etoposide and doxorubicin, two chemotherapeutic drugs, as well as lenvatinib were utilized to determine the effect of NOX4 on drug resistance. Lenvatinib-resistant BCPAP cells (LRBCs) were established to confirm this effect. The underlining mechanisms of NOX4 under starvation were explored using western blot. Finally, GLX351322, an inhibitor targeting NOX4, was used to inhibit NOX4-derived ROS in vitro and detect its effect on drug resistance of tumor cells in vivo. NOX4 is overexpressed under serum deprivation in BCPAP or TPC-1 cells. NOX4 knockdown impairs cell viability, increases cell apoptosis, extends G1 phase during cell cycle and modulates the level of energy-associated metabolites in starved cells. When the starved cells or LRBCs are treated with chemotherapeutic drugs or Lenvatinib, NOX4 knockdown inhibits cell viability and aggravates cell apoptosis depending on NOX4-derived ROS production. Mechanistically, starvation activates TGFβ1/SMAD3 signal, which mediates NOX4 upregulation. The upregulated NOX4 then triggers ERKs and PI3K/AKT pathway to influence cell apoptosis. GLX351322, a NOX4-derived ROS inhibitor, has an inhibitory effect on cell growth in vitro and the growth of BCPAP-derived even LRBCs-derived xenografts in vivo. These findings highlight NOX4 and NOX4-derived ROS as a potential therapeutic target in resistance to PTC.

## Introduction

Papillary thyroid carcinoma (PTC) is the most frequent histologic type of thyroid cancer, accounting for greater than 80% of cases [1]. The 10-year survival rate among patients with PTC that is refractory to radioiodine therapy is 10% from the time of detection of metastasis [2–4]. It has been reported that elevated levels of reactive oxygen species (ROS) as a risk factor for the development of PTC in patients with Hashimoto thyroiditis [5], and that increased aerobic glycolysis portends an unfavorable prognosis in follicular thyroid cancer [6]. Thus, it can be reflected that ROS level and glycolytic activity can be proposed as risk factors for the development of PTC.

Cells produce ROS through a variety of enzymatic systems, including NADPH oxidases (NOXs), mitochondrial electron transport chain, et al. [7]. The involvement of NOXs, which produce ROS as their primary and sole function, has become of particular interest in thyroid malignancy [8]. Among the NOXs, NOX4 was demonstrated to be upregulated in thyroid cancers such as PTC, so did its binding partner, p22phox [9, 10]. In a BRAF-mutant PTC cell line, NOX4-dependent ROS generation was involved in BRAF^V600E^-induced NIS (sodium/iodide symporter) repression via TGF-β/Smad3 signaling pathway [11]. In renal cancer which more readily uses glycolysis, NOX4 acts as a mitochondrial energetic sensor to modulate ATP levels in providing hope for overcoming drug resistance when combined with cytotoxic drugs [12]. Furthermore, we previously reported that NOX4 sustains cell growth in hypoxic PTC via ROS-HIF-glycolysis pathway [10]. Based on the effect of NOX4 on the pathway and drug resistance, we wanted to address the role of NOX4 in resistance of PTC cells.

Lenvatinib has been approved by the US Food and Drug Administration as a tyrosine kinase inhibitor (TKI) for radioiodine-refractory differentiated thyroid cancer [13]. The approval was based on a study of 392 people with DTC that grew after they received radioactive iodine therapy. Patients in the study who received lenvatinib lived an average 18.3 months before their cancer started growing again compared to an average 3.6 months for patients who did not receive lenvatinib [14], demonstrating lenvatinib as a multitargeted TKI improved median progression-free survival in patients with thyroid carcinomas. However, lenvatinib has no significant impact on improving the overall survival rate for this disease so almost 50% of patients with advance DTC treated with lenvatinib will develop significant disease progression after average of 18 months due to resistance mechanisms [15–17]. Importantly, lenvatinib could induce elevated ROS production [18], and resistance to lenvatinib leads to increased glycolysis [16]. Combining with the role of NOX4 in ROS and glycolysis in PTC [10], it still needs to be further investigated whether NOX4 is involved in the process of resistance to lenvatinib and how.

## Results

### NOX4 is required for cell survival of starved PTC cells

**Fig. 1:**
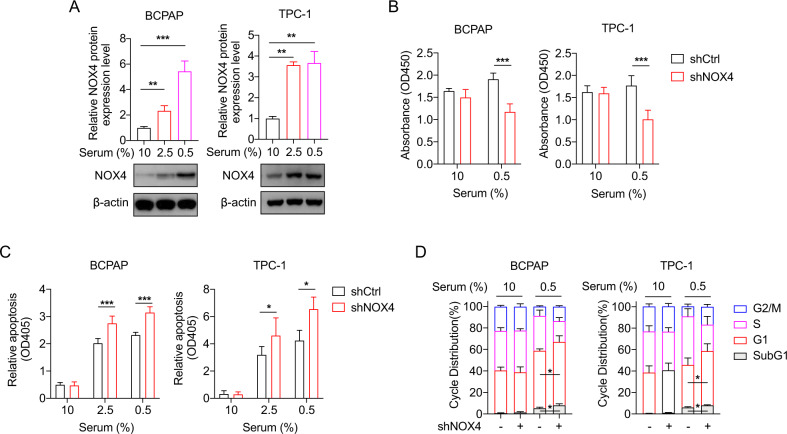
The role of NOX4 in cell biology in starved PTC cells. **A** Quantification of NOX4 protein by immunoblot in BCPAP and TPC-1 cells, which were cultured in 1640 and DMEM medium respectively supplemented with 10%, 2.5%, or 0.5% FBS for 48 h. n = 4. **B** Cell viability was assessed in the indicated PTC cells expressing shRNA against NOX4 or not, which were cultured under 10% or 0.5% FBS for 48 h by CCK-8 assay. *n* = 5. **C** Cell apoptosis was assessed in the indicated cells expressing shRNA against NOX4 or not, which were cultured under 10%, 2.5%, or 0.5% FBS for 48 h by the Cell Death Detection ELISAPLUS kit. *n* = 3. **D** Distribution of different phases during cell cycle in the indicated cells expressing shRNA against NOX4 or not under 10% FBS or 0.5% FBS condition for 48 h (subG1, events with a < 2 N DNA content). *n* = 3. Values are expressed as the means ± SD of biological replicates. *P < 0.05, **P < 0.01, ***P < 0.001.

Previously we reported that NOX4 is sensitive to hypoxia [10]. Since serum starvation is a common feature of solid tumors like hypoxia [20], we wonder whether NOX4 is reactive to serum starvation. Immunoblots demonstrated that once the serum concentration was decreased from 10% to 0.5%, NOX4 expressions in BCPAP and TPC-1 cells were significantly increased (Fig. 1A), showing its starvation-reactive character. Then the role of NOX4 in cell viability and apoptosis under starvation was investigated. The cells infected with lentivirus expressing shRNA against NOX4 (shNOX4) or control shRNA (shCtrl) were conducted with a CCK8 assay (Fig. S1A). The results showed that NOX4 knockdown significantly represses cell viability under starvation (Fig. 1B). Cell death assays detected by Cell Death Detection ELISAPLUS kit showed a significant increase in apoptosis in NOX4-deficient cells under starvation (Fig. 1C). Cell cycle assays showed that under starvation NOX4 knockdown induces a significantly extended G1 phase under starvation (Fig. 1D). The increase of apoptotic sub G1 fraction was consistent with the alteration of apoptosis in Fig. 1C. Altogether, these results underscore the importance of NOX4 in determining cell survival in starved PTC cells.

### NOX4 deficiency modulates the levels of energy-associated metabolites of BCPAP cells

To get metabolic insights of NOX4 in starved PTC cells, the targeted metabolomics analyses by mass spectrometry were conducted upon starvation or not. The principal component analysis demonstrated a distinct separation between shCtrl and shNOX4 cells under 10%- and 0.5%- serum (Fig. S2A). Heat maps further showed that the level of each metabolite was inconsistently altered by NOX4 deficiency (Fig. S2B). In detail, the lactate level was reduced significantly (Fig. S2C), but the rations of ADP/ATP, AMP/ATP, and NAD+/NADH were significantly increased in NOX4-deficient BCPAP cells under serum-starved condition (Fig. S2D, E), which gives us a clue that NOX4 may involve in glycolysis, the energy demand, and redox regulation under starvation.

### NOX4 depends on ROS to oppose chemotherapeutic drugs-induced apoptosis

**Fig. 2:**
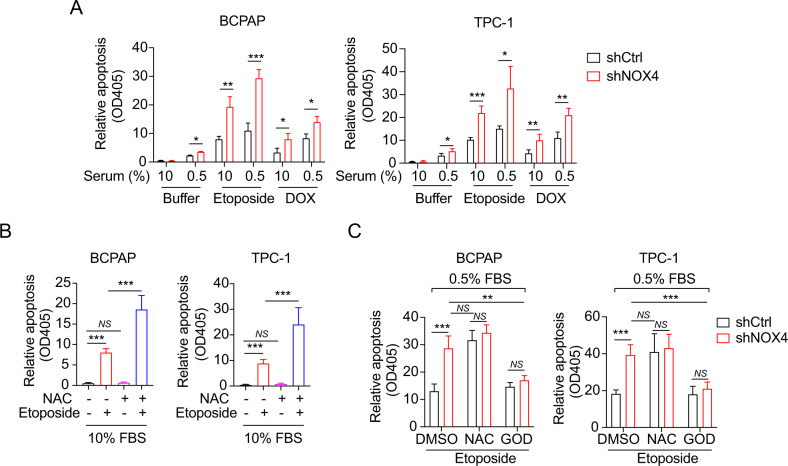
NOX4 attenuates apoptosis of starved PTC cells in response to chemotherapeutic drugs through NOX4-derived ROS. **A** Cell apoptosis was assessed by the Cell Death Detection ELISAPLUS kit. BCPAP or TPC-1 cells exposed to etoposide (20 μM) or doxorubicin (1 μg/ml) under 10% or 0.5% serum condition. *n* = 3. **B** Cell apoptosis was assessed in the indicated PTC cells cultured under 10% FBS for 48 h and treated with the combination of NAC and Etoposide or each alone. *n* = 3. **C** Cell apoptosis was assessed in the indicated PTC cells exposed to DMSO or NAC (10 mM) or GOD (100 μg/mL) at a serum concentration of 0.5%. *n* = 3. Values are expressed as the means ± SD of biological replicates. *P < 0.05, **P < 0.01, ***P < 0.001.

Given that NOX4 functions as an energetic sensor coupling cancer metabolic reprogramming to drug resistance [12], we then assumed that NOX4 contributes to the acquisition of drug resistance in starved PTC cells. After the cells were treated with chemotherapeutic drugs (etoposide and doxorubicin (DOX)), we found that the cells exposed to etoposide or DOX reveal a normal enhancement of cell apoptosis compared to the buffer alone, whereas the starved cells showed a modest increase of the drugs-induced cell death (Fig. 2A). More importantly, there was also a significant increment of apoptosis in NOX4-deficient cells compared with their controls (Fig. 2A), which supports an inhibitory role of NOX4 in the chemotherapeutic drugs-elicited cell death.

We then asked whether the effect of NOX4 on drug-induced cell death is mediated by ROS. The PTC cells were treated with N-Acetyl-L-cysteine (NAC), a ROS scavenger, and etoposide respectively or combinedly in completed medium. The results showed that NAC cannot elicit additional apoptosis but does enhance etoposide-induced apoptosis (Fig. 2B). Under starvation, combining NAC with etoposide leads to more rates of apoptosis than etoposide alone, almost equaling the level caused by shNOX4 alone, whereas combining NAC plus shNOX4 was disabled to aggravate etoposide-induced apoptosis compared with the level caused by shCtrl plus NAC (Fig. 2C). This indicated that NAC alone in the presence of etoposide increases apoptosis under starvation, NOX4 knockdown does not change the effect of NAC, and cell survival under starvation and etoposide treatment depends on NOX4-derived ROS production. Furthermore, these findings can be confirmed reversely by the addition of glucose oxidase (GOD), a ROS generator (Fig. 2C).

To directly evaluate the effects of NOX4 on ROS production, we constructed three NOX4 expression vectors, named WT, RRE, and 41b. WT expresses a wild-type form of NOX4, whereas RRE and 41b are mutated, inactive, competitive forms of NOX4 as dominant-negative phenotypes (Fig. S3A) [19]. Immunoblots showed that WT leads to an overexpression of NOX4 protein, so do RRE and 41b despite their mutants (Fig. S3B). DCFDA staining demonstrated that starvation induced a substantial increase of ROS, and WT can further significantly elevate the ROS level, whereas the two mutants significantly declined the ROS level under starvation (Fig. S3C, D). The shNOX4 can also confirmed this inhibitory effect of the mutants on ROS production (Fig. S3E, F). These findings highlight a positively regulatory impact of NOX4 on total ROS level produced by PTC cells after serum starvation.

### NOX4 is required for PTC cell survival in response to lenvatinib depending on NOX4-derived ROS

**Fig. 3:**
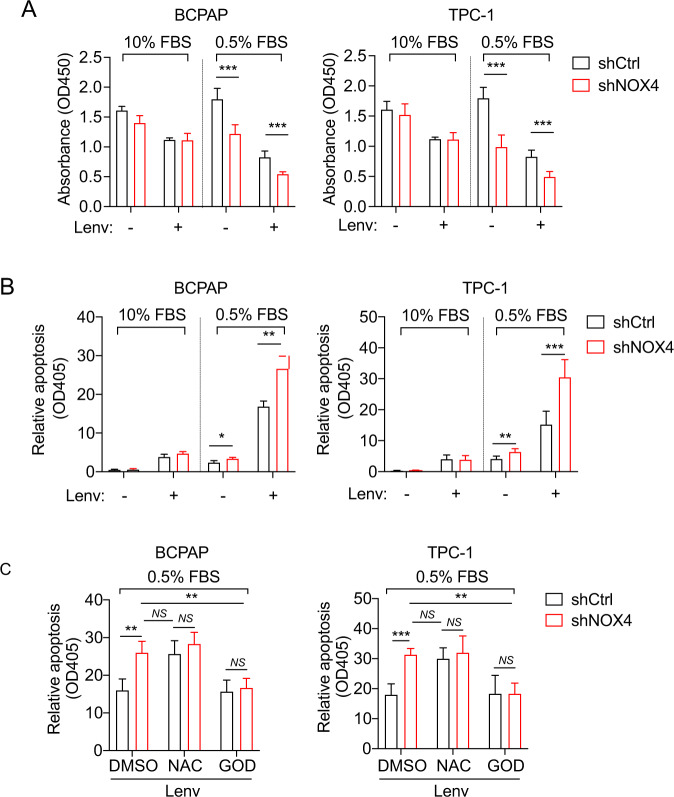
NOX4 and NOX4-derived ROS is required for survival of starved PTC cells treated with lenvatinib. **A** Cell viability was assessed in the indicated PTC cells cultured under 10% or 0.5% FBS for 48 h and treated with DMSO or lenvatinib (3 μM) by CCK-8 assay. *n* = 3. **B** Cell apoptosis was assessed in the indicated PTC cells exposed to DMSO or lenvatinib (3 μM) at a serum concentration of 10% or 0.5%. *n* = 3. **C** Cell apoptosis was assessed in the indicated PTC cells exposed to DMSO or NAC (10 mM) or GOD (100 μg/mL) at a concentration of 3 μM Lenvatinib under 0.5% FBS condition. *n* = 3. Values are expressed as the means ± SD of biological replicates. *P < 0.05, **P < 0.01, ***P < 0.001. NS, no significance.

Lenvatinib, a multi-targeted anticancer agent for differentiated thyroid cancer [13], was then utilized in our system. A CCK8 assay demonstrated that lenvatinib obviously decrease cell viability under normal and starved conditions, but NOX4 knockdown can further significantly reduce cell viability under starvation (Fig. 3A). Correspondingly, apoptotic assays showed that the lenvatinib-treated cells expressing shNOX4 under starvation has a higher apoptotic rate than their controls (Fig. 3B). Interestingly, it seems that the level of cell death induced by lenvatinib with 10% serum does not depend on NOX4, different from etoposide and DOX. This may be due to the fact that lenvatinib at 10% serum triggers apoptotic signals different from these drugs. Likewise, we used NAC and GOD to determine whether the role of NOX4 in lenvatinib-induced cell death under starvation is mediated by NOX4-derived ROS. The results revealed that neither NAC nor GOD shows significance between NOX4 knockdown and its control, whereas NOX4 knockdown increases significant induction of apoptosis without NAC or GOD (Fig. 3C). These data confirmed that NOX4 depends on ROS to regulate lenvatinib-induced apoptosis under starvation.

### The survival of LRBCs requires NOX4 and NOX4-derived ROS

**Fig. 4:**
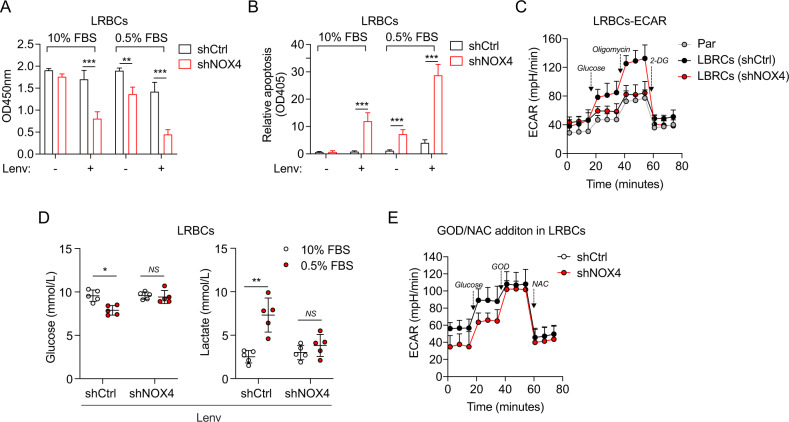
NOX4 deficiency weakens the resistance of LRBCs to Lenvatinib through NOX4-derived ROS. **A** Cell viability was assessed by CCK8 assay in LRBCs cultured under 10% or 0.5% FBS for 48 h and treated with DMSO or lenvatinib (10 μM). *n* = 3. **B** Cell apoptosis was assessed in LRBCs cultured under 10% or 0.5% FBS for 48 h and treated with DMSO or lenvatinib (10 μM). *n* = 3. **C** The ECAR analysis in the parental LRBCs (BCPAP cells) or LRBCs expressing shRNA against NOX4 or not. *n* = 3. **D** Supernatants of LRBCs expressing shRNA against NOX4 or not under 10% FBS or 0.5% FBS at a concentration of 10 μM Lenvatinib for 48 h were analyzed for glucose and lactate concentrations. *n* = 3. **E** The modified ECAR analysis in LRBCs expressing shRNA against NOX4 or not. *n* = 3. Values are expressed as the means ± SD of biological replicates. *P < 0.05, **P < 0.01, ***P < 0.001. NS, no significance.

We afterward characterized lenvatinib-resistant BCPAP cells (LRBCs) that had been exposed to doses of lenvatinib increased by 0.125 μM per week and until reaching a final concentration of 32 μM. IC50 of parent cells treated with lenvatinib in 72 h was 2.91 μM, while those of LRBCs were 10.74 μM (Fig. S4A), suggesting that LRBCs successfully acquired lenvatinib resistance. Then the NOX4 expression was still knocked down by lentivirus-mediated shRNA. Immunoblots confirmed the NOX4 protein expression in LRBCs and NOX4-deficient LRBCs (Fig. S4B). The CCK8 and apoptosis assays together showed that NOX4 knockdown significantly inhibits cell survival and enhances cell apoptosis of lenvatinib-treated LRBCs independent of starvation (Fig. 4A and B). Of note, starvation tends to break the resistance of LRBCs to lenvatinib, but NOX4 deficiency aggravates the break of resistance.

Resistance to lenvatinib increases glycolysis [16], thus we wanted to assess the effect of NOX4 on glycolysis in LRBCs. The measurements of the extracellular acidification rate (ECAR) showed that LRBCs have a higher glycolytic capacity than the parent cells, but the increment can be dropped close to the level caused by shNOX4 in the parent cells (Fig. 4C). Since the start and end ports during glycolysis are represented by glucose uptake and lactate secretion, we investigated the influence of NOX4 on glucose uptake and lactate secretion in LRBCs. As expected, starvation decreased glucose levels and increased lactate levels in lenvatinib-treated LRBCs, whereas these differences were disappeared in NOX4-deficient LBRCs (Fig. 4D), supporting an indispensable role of NOX4 in the start and end port of glycolysis under starvation in lenvatinib-resistant cells.

Next, we performed modified assay to identify whether ROS mediates the role of NOX4 in the glycolysis of LRBCs by sequential injection with GOD and NAC post glucose. The results demonstrated that NOX4 knockdown leads to the downregulation of the basal glycolysis until the injection of GOD that rescues the effect of NOX4 deficiency, and both increments of ECAR were blocked by the addition of NAC (Fig. 4E). Collectively, these findings suggest that NOX4 is required for cell survival of lenvatinib-resistant PTC cells and highlight a critical role of NOX4-derived ROS in glycolytic activity.

### GLX351322 leads to decreased cell survival of PTC cells and LRBCs

**Fig. 5:**
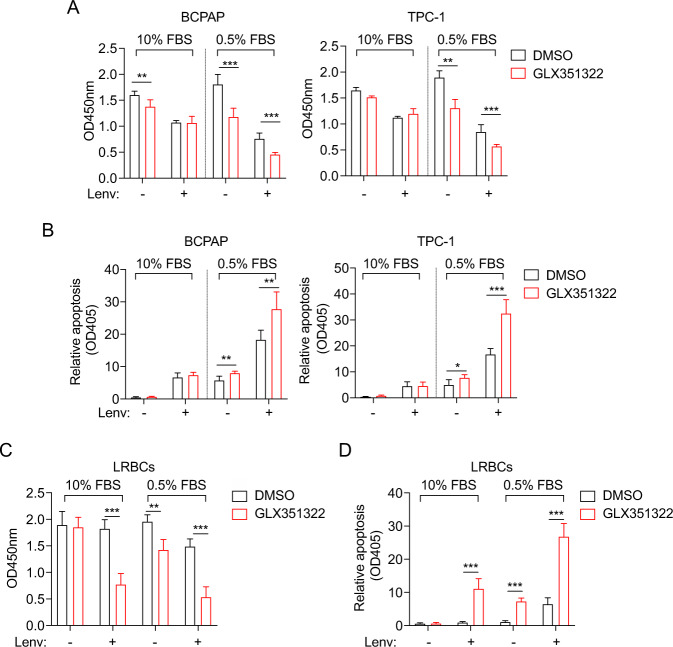
GLX351322 enhance cell death of PTC cells and LRBCs treated with lenvatinib. **A** Cell viability was assessed in the indicated PTC cells cultured with medium containing 10% or 0.5% FBS, and DMSO or GLX351322 (5 μM) for 48 h by CCK-8 assay. *n* = 3. **B** Cell apoptosis was assessed by the Cell Death Detection ELISAPLUS kit using the indicated PTC cells cultured in medium containing 10% or 0.5% FBS, and DMSO or GLX351322 (5 μM) for 48 h. *n* = 3. **C** Cell viability was assessed in LRBCs cultured with medium containing 10% or 0.5% FBS, and DMSO or GLX351322 (5 μM) for 48 h by CCK-8 assay. *n* = 3. **D** Cell apoptosis was assessed using LRBCs cultured in medium containing 10% or 0.5% FBS, and DMSO or GLX351322 (5 μM) for 48 h. *n* = 3. Values are expressed as the means ± SD of biological replicates. *P < 0.05, **P < 0.01, ***P < 0.001. NS, no significance.

Since NOX4-derived ROS is vital for cell survival in starved PTC cells, we next used a chemical inhibitor targeting NOX4, GLX351322 (GLX) [21], to inhibit NOX4-derived ROS. As expected, GLX significantly reduced cell viability under starvation independent of lenvatinib (Fig. 5A), which suggests that the mechanisms underlining GLX are not consistent with lenvatinib. This also indicate that the combination of both drugs may have a satisfied antitumor effect in vivo. The apoptotic assays also confirmed this effect (Fig. 5B). In LRBCs, GLX significantly decreased the survival ratios in lenvatinib-treated cells independent of starvation or without lenvatinib under starvation (Fig. 5C), revealing that the combinatory usage of lenvatinib and GLX can maximize the inhibitory effect. A similar trend was confirmed in the detection of apoptosis in LRBCs as well (Fig. 5D). These findings suggests that GLX can effectively break the resistance of PTC cells to lenvatinib.

### The impact of inhibiting NOX4-derived ROS by inhibitors or dominant-negative mutants on the ROS levels

The impact on ROS level of inhibiting NOX4 with GLX or with NAC was assessed. The results were presented in Fig. S5A–D and can be summarized as follows: (1) substantial reductions of ROS level under starvation were induced by either GLX351322 or NAC; (2) NAC has a better inhibitory effect on ROS level than GLX and NOX4 (Figs. S5A–D and S3E, F). These data not only indicate that NOX4 has an obviously positive impact on ROS production under starvation, but also reveal that NOX4-mediated ROS production is one of the major cellular sources but not all under starvation.

We wanted to further find out whether the NOX4 mutants have a better effect on inhibiting ROS than GLX or shNOX4. The results showed that the NOX4 mutants can further lead to a significant reduction of ROS level even in the presence of shNOX4 or GLX351322, but they failed to induce any significant changes in the presence of NAC (Fig. S5E, F). This indicates that the NOX4 mutants do have a better effect on ROS inhibition than shRNA and GLX with the exception of NAC, and that in addition to NOX4 there are other signals responsible for ROS generation under starvation.

### Upregulation of NOX4 under starvation is mediated by TGFβ1/SMAD3 signal and triggers ERKs and PI3K/AKT pathway

**Fig. 6:**
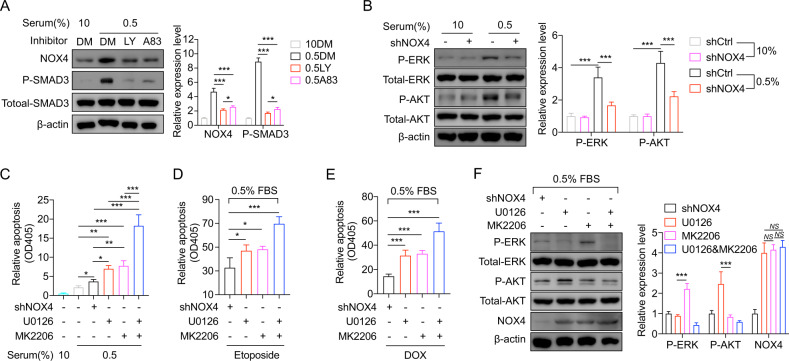
Upregulation of NOX4 upon starvation is mediated by TGFβ1/SMAD3 signal and triggers ERKs and PI3K/AKT pathway. **A** Immunoblots indicating NOX4 and phosphorylated SMAD3 protein expression in BCPAP cells treating with starvation and the indicated inhibitors. *n* = 3. DM, DMSO. LY, LY2157299 (50 μM). A83, A83-01 (10 μM). **B** Immunoblots showing phosphorylated ERK and phosphorylated ERK expression in BCPAP cells expressing shRNA against NOX4 under starvation. *n* = 3. **C**–**E** Cell apoptosis was assessed in BCPAP cells treating with starvation or the indicated shRNA, U0126 (20 μM), MK2206 (2.5 μM), etoposide (20 μM) or doxorubicin (1 μg/ml). *n* = 4. **F** Immunoblots demonstrating NOX4 and phosphorylated ERK and phosphorylated ERK expression in BCPAP cells expressing shRNA treated with U0126 alone, (20 μM) MK2206 alone (2.5 μM), or the combination. *n* = 3. Values are expressed as the means ± SD of biological replicates. *P < 0.05, **P < 0.01, ***P < 0.001. NS, no significance.

We next investigate how NOX4 is upregulated after serum deprivation. The main inducer of NOX4 expression is TGF-β [22, 23]. Therefore, the phosphorylation of SMAD3 on Ser423/425 were detected to assess the status of TGF-β pathway. Immunoblots showed that the phosphorylation of SMAD3 is enhanced under starvation (Fig. 6A), demonstrating that TGF-beta pathway is activated by starvation. Galunisertib (LY2157299) [24, 25] and A83-01 [26], two TGFβ1 receptor inhibitors, were utilized to block this pathway. It was found that the upregulation of NOX4 after serum deprivation is significantly diminished (Fig. 6A), which demonstrates that the upregulation of NOX4 after serum deprivation depends on TGFβ1/SMAD3 signal transduction.

ERKs or PI3K/AKT signals in thyroid cancer are critical [27, 28], we hypothesized that ERKs or PI3K/AKT as downstream survival signals are activated by NOX4. As expected, immunoblots exhibited that the phosphorylation of ERK and AKT was not altered under normal condition; whereas under starvation the phosphorylated AKT and phosphorylated ERK levels were increased, but these increased levels can be partly blocked by interfering NOX4 (Fig. 6B). This indicates both ERKs and PI3K/AKT are regulated by NOX4 under starvation. U0126 and MK2206 are MEK/ERK-specific and AKT-specific inhibitors, respectively [29, 30]. Apoptotic assays showed that under starvation either U0126 or MK2206 leads to a significant enhancement of apoptosis compared to shCtrl just like the situation caused by shNOX4 (Fig. 6C). When treating starved cells with etoposide or DOX, it also can be observed that either U0126 or MK2206 has a superior effect over shNOX4 on cell apoptosis (Fig. 6D, E), confirming the importance of ERKs or PI3K/AKT as downstream of NOX4 under these conditions. Notably, combining U0126 with MK2006 exhibited a better synergic effect on apoptosis than either treatment alone (Fig. 6C–E), indicating the existence of the crosstalk and independence between the two pathways under these conditions.

The crosstalk of the two downstream pathways was further investigated. Immunoblots showed that inhibition of a single arm of either AKT or ERK pathway using MK2206 or U0126 would induce the increase of another arm, and that the combination reduces the phosphorylated ERK or AKT to a level lower than shNOX4 (Fig. 6F). On another side, based on the unaltered expression of NOX4, it can be concluded that either ERK or AKT signal has no obvious impact on NOX4 expression. These findings are consistent with the conclusions in previous reports [31–33].

### Combination of GLX351322 and lenvatinib completely suppresses PTC tumor growth even in LRBCs

**Fig. 7:**
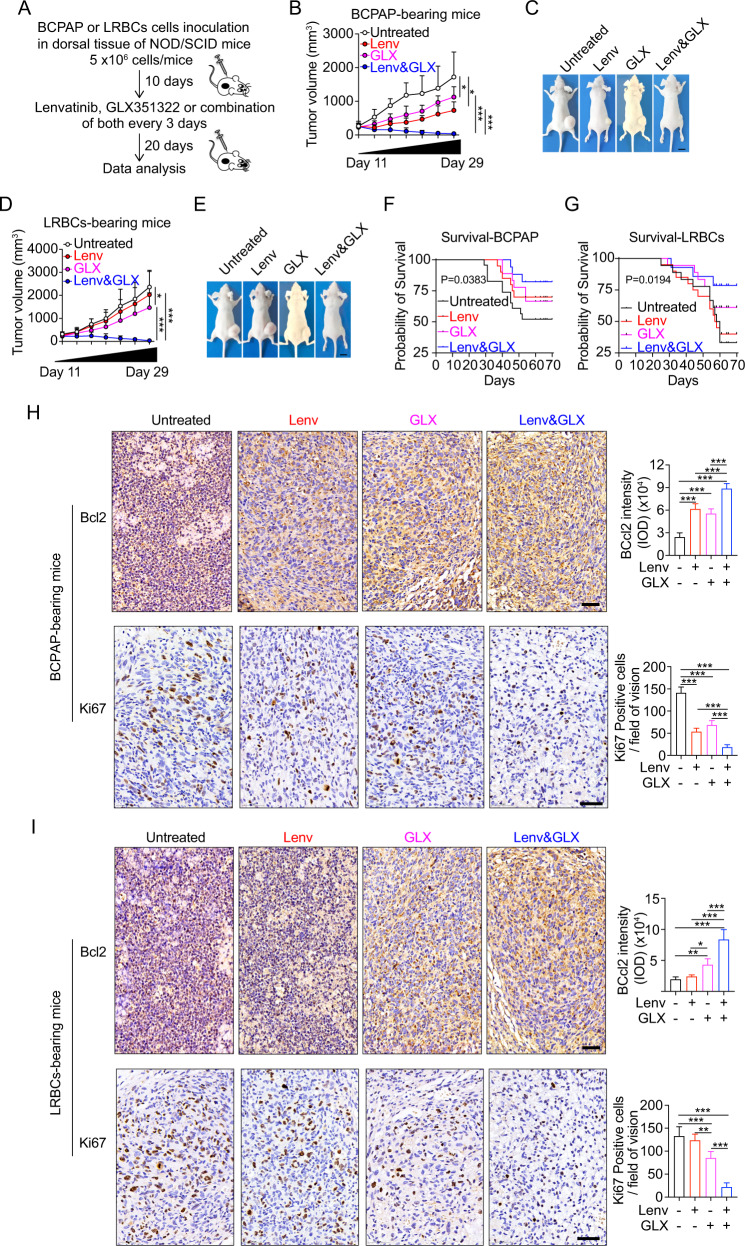
Lenvatinib in combination with GLX351322 completely suppresses PTC tumor growth. **A** Schematic diagram of inoculation and therapy in a mouse xenograft model. Growth curve or representative gross morphology of xenografts on mice bearing BCPAP cells (**B**, **C**) or LRBCs (**D**, **E**) in dorsal tissues for 10 days were left untreated or were treated with lenvatinib (30 mg/kg), GLX351322 (5 mg/kg), or lenvatinib plus GLX352322 as described. *n* = 5. **F**–**G** Kaplan-Meier survival curve showing significant improvement in survival of the mice treated with the combined drug as compared with single drug-treated mice for 70 days after inoculation. **H**–**I** IHC staining of Bcl2 or Ki67 in BCPAP- or LRBCs- bearing mice. Values are expressed as the means ± SD of biological replicates. *P < 0.05, **P < 0.01, ***P < 0.001. NS, no significance.

We further constructed BCPAP- or LRBCs- bearing mice to assess the effect of lenvatinib alone, GLX351322 alone, or the combination of both (Fig. 7A). Lenvatinib or GLX351322 partially affected the progression of BCPAP-derived tumors, but the combination significantly reduced tumor volumes, eventually eliminating the tumors in vivo (Fig. 7B, C). Analogously, the combination completely suppresses the LRBCs-derived tumors, although lenvatinib alone has no significantly inhibitory effect on LRBCs-bearing tumors (Fig. 7D, E). A Kaplan–Meier survival curve showed significantly better survival of the mice treated with the combined drug (Fig. 7F, G). The apoptosis and proliferation of both the BCPAP- and LRBCs- bearing tumors after double treatment with lenvatinib and GLX351322 were then analyzed by immunohistochemistry. The results showed that in BCPAP-bearing tumors either lenvatinib or GLX351322 treatment alone increases Bcl2 expression and reduces Ki67-positive cells, and the combination of both drugs can further enhance this effect (Fig. 7H), characterizing a higher apoptosis and a lower proliferation upon the drugs treatment. In the LRBCs-bearing tumors, lenvatinib has no significantly inhibitory effect on Bcl2 and Ki67 expression, but GLX351322 alone and the combination can significantly increase Bcl2 expression and reduces Ki67-positive cells (Fig. 7I). The combination has a superior effect than GLX351322 alone. These findings signify the importance of NOX4 and the clinical significance of the combination against resistance to lenvatinib in PTC tumors. Of note, we also found that NOX4 expression positively correlated with HIF1α (resistant marker), Glut1 and LDHA (glycolytic markers), and Ki67; whereas it negatively correlated with Bcl2 in tumor tissues in patients with PTC (Fig. S6A). Taken together, these data demonstrate that GLX351322 exerts a suppressive effect on PTC tumor growth, and combining GLX351322 with lenvatinib can completely suppress PTC tumor growth even in LRBCs.

## Discussion

TPC-1 and BCPAP cells are BRAF non-mutated and mutated cell lines respectively, but in the present study we did not evaluate the impact of BRAF mutation on NOX4 experimentally. Indeed, NOX4 expression levels are significantly increased in PTCs with a BRAF mutation in TCGA data [34]. Further study showed that NOX4 upregulation is controlled at the transcriptional level by the BRAF^V600E^ oncogenic protein via the TGF-β/Smad3 signaling pathway dependent of MEK/ERK, and that knockdown of NOX4 downregulates BRAF^V600E^ -induced NIS repression [11]. A successful treatment for thyroid cancer is strictly correlated with an active Sodium Iodide Symporter (SLC5A5; NIS), which allows the retention of radioiodine in the tumor cells [35]. Based on these reports, it can be concluded that NOX4 expression is closely linked to BRAF mutant as shown exactly as BRAF^V600E^-MEK-TGF-β/Smad3-NOX4-ROS-SLC5A5 (NIS) axis. Consequently, NOX4 is involved in the absence of radioiodine uptake by BRAF^V600E^ PTC cells. While in our present study, the effect of NOX4 knockdown on cell viability, cell apoptosis, and cell cycle is likely to be independent of BRAF mutant, because all results of NOX4 knockdown in both cell lines harboring BRAF mutant or not present similar tendency. In other words, BRAF mutant may not be involved the signaling of NOX4 in drug resistance of PTC cells. Consistently, we showed that upregulation of NOX4 under starvation is mediated by TGFβ1/SMAD3 signal and triggers ERKs and PI3K/AKT pathway.

Tumor cells including PTC cells face challenging physiological conditions, including hypoxia, low nutrient availability, and exposure to therapeutic drugs throughout their progression from tumor precursors to metastases [36]. They have to adapt to nutrient-deprived conditions for survival. Serum starvation resembles the growth factor deprivation characteristic of the poorly vascularized tumor microenvironment [37, 38]. This condition induces cell cycle synchronization arresting at the G1 phase [39] and increases apoptosis [40]. Our results in this study are in line with these previous studies, further provide novel insights into the mechanisms underlying NOX4-driven adaptations to serum starvation. It has been also reported that serum starvation increases stemness of cancer cells [39], thus it can be concluded that NOX4 may involve multiple alterations for adaptation to serum starvation, not even limited to this stress. Further investigation would be required to expand our understanding of NOX4-responsive stresses among various tumor microenvironments and elucidate the detailed mechanisms of adapting to these stresses.

Lenvatinib can suppress DTC albeit existence of adverse events [41], but tumor cells developed resistant mechanisms that involved alternative pathways for survival [42]. Metabolically, lenvatinib resistance leads to increased glycolysis [16], so overcoming this increase would be a potential strategy to solve the resistance. Previously we reported that NOX4 serves as a glycolytic regulator via ROS in hypoxia [10], the present study demonstrated not only the supportive effect of NOX4 on lenvatinib-induced glycolysis under serum-starved conditions via ROS in PTC cells in vitro, but also the surprising effect upon combinatory treatment of GLX351322 and lenvatinib in vivo, suggesting that PTC cells depend on NOX4 or NOX4-derived ROS to establish plasticity that is capable of responding to complex and changeable tumor microenvironments. So far, the understanding of NOX4 in tumor microenvironments remains fragmentary, but it can be inferred that NOX4 is required for microenvironments-stressed PTC cells that need ROS and glycolysis to serve as the major bioenergetic pathway. In future it is a challenge to develop new drugs associated with NOXs-derived ROS or drug combinations with less adverse effect and improve antitumor activity based on these findings.

## Methods

### Cells and cell counting Kit-8

The human papillary thyroid carcinoma cell lines BCPAP and TPC-1 were cultured in RPMI 1640 medium (Thermo fisher, 11875168) supplemented with 10% fetal bovine serum (FBS) (Thermo fisher,10437-028) and antibiotics in a 37 °C incubator with 5% CO_2_. All cell lines were authenticated using STR profiling and tested negative for mycoplasma contamination. For serum starvation, cells were cultured medium supplemented with 0.5% FBS. The experiment was replicated three times.

### Cell viability

Cell viability was determined by Cell Counting-Kit 8 assay (MCE), which was performed in 96-well plates and the absorbance was measured at 450 nm in a microplate reader (Thermo Varioskan Flash) after 2 h of incubation. The assays were performed with *N* = 4 and repeated three times. The experiment was replicated three times.

### Cell apoptosis

Cell apoptosis was measured using the Cell Death Detection ELISAPLUS kit (MERCK, CAT #11774425001) following the manufacturer’s protocol. The experiment was replicated three times.

### Cell cycle

The cells (1 × 10^6^) were fixed with 70% ethanol for 1 h at room temperature (25 °C). The samples were then centrifuged at 1000 *g* for 5 min. The 70% ethanol was removed, and the cells were then treated with 100 μL of RNase A (0.5 mg/ml) for 30 min at 37 °C. Cell samples were then stained with 20 μg/ml of PI and analyzed with a BD FACSCalibur flow cytometer to obtain DNA content profiles. FlowJo 10.0 software was used for analysis of the cell cycle. The experiment was replicated three times.

### ROS determination

Detection of cellular ROS using DCFDA (20 µM; Abcam) was performed by incubating cells in culturing medium 1 h at 37 °C and measuring by flow cytometry (BD LSR Fortessa). Data were then analyzed with FlowJo 10.4. The experiment was replicated three times.

### Glucose and Lactate quantification

Glucose and lactate in supernatants of LRBCs expressing shNOX4 or shCtrl under 10% or 0.5% FBS were measured turbidimetrically on a Cobas8000 (Roche). Briefly, cells were divided into four groups: shCtrl cultured in 10% FBS, shCtrl cultured in 0.5% FBS, shNOX4 cultured in 10% FBS, and shNOX4 cultured in 0.5% FBS. Each group had five biological replicates. 500,000 cells were seeded into each well of 6-well plates, and cultured in RPMI 1640 medium that contains 10 μM lenvatinib, 2000 mg/L D-glucose, and none of the lactate. After 48 h, these supernatants were respectively collected and analyzed quantitatively by instrument. The experiment was replicated three times.

### Plasmid and lentivirus production

Plasmids (pLKO.1) encoding for scrambled shRNA and human NOX4 shRNA were purchased from MISSION shRNA (Sigma-Aldrich). The shRNA targeting sequence was 5ʹ- GCTGT ATATT GATGG TCCTT T-3ʹ. Lentivirus was packaged in 293T cells using psPAX2 and VsvG, followed by viral transduction to BCPAP cells or TPC-1 cells with 5 µg/µl polybrene. Twenty-four hours post-infection, single cells were selected and passed into 96-well plates in puromycin (1000 ng/ml) antibiotic for selection and maintained in puromycin (500 ng/ml). Plasmid pcDNA3.1 was used to construct the NOX4 wild type and mutants including WT, RRE, and 41b. The sequences were obtained from published reference [19]. Primers and genes were synthesized by Tsingke Biotech (ChengDu). Then human NOX4 cDNA with mutant or wild type was cloned into the linearized vector using seamless clone kit.

### Targeted metabolomics

BCPAP cells stably expressing shCtrl and shNOX4 in triplicate under 10%- or 0.5%- serum condition for 48 h were mixed with cold methanol/acetonitrile/H_2_O (2:2:1, v/v/v) to remove the protein. Then all samples were sent to ShangHai Applied Protein Technology for further sample preparation and mass spectrometry analysis.

### Xenografts

All animal experiments were approved by the Ethics Committee of the Third Hospital of Mianyang and the Ethics Committee for Animal Research BEIJING VIEWSOLID BIOTECH CO.LTD. Mouse xenografts models were established by subcutaneous injection into the flanks of NOD/SCID mice (6–9 weeks-old females, Charles River) with 5 × 10^6^ BCPAP cells or LRBCs. When tumors grew to about 200–400 mm^3^ after 10 days, the mice were randomly separated into four groups, one for water (untreated), one for GLX351322 (5 mg/kg, Selleck, CAT #S0178), one for lenvatinib (30 mg/kg, Selleck, CAT #S1164), and one for the combination of GLX351322 and Lenvatinib. All drugs were administered orally once per three days. There were at least 5 mice in each group. Tumor size was measured every 3 days, and the tumor volume was calculated as Volume = Length × Width × Width × 0.52. To evaluate the survival rate, the number of mice in each group was increased up to no less than 15. Then these mice were monitored for 70 days. All inclusion/exclusion criteria were pre-established and no samples or animals were excluded from the analysis. During the experiments, the investigator was not blinded to the group allocation, either while doing the experiment or while assessing the results.

### IHC staining

The tumor tissues derived from xenograft at almost 25–29 days post inoculation were carefully stripped off and were fixed for 24 h in 10% formalin. Then the samples were embedded in paraffin. Antigen unmasking was achieved by placing the slides in a microwave oven on high for 10 min in 10 mM sodium citrate buffer (pH 6.0), followed by a 20 min cooldown at room temperature. After a blocking step with 5% goat serum and Avidin-Biotin blocking kit, the slides were incubated with primary antibodies overnight at 4 °C. Primary antibodies used were Anti-Bcl2 (Proteintech, CAT #26593-1-AP) and anti-Ki67 (Abcam, CAT #ab156956). Slides were then subjected to 3% hydrogen peroxide for 10 min to quench endogenous peroxidase activity. Image Pro Plus 7 software was used for quantification.

### Western blotting

The cells that stably expressing shNOX4 or shCtrl were harvested when the cell number was almost 1 million cells per well in six-well plate by microscopical evaluation. Then the cells were lysed in RIPA buffer (MERCK, CAT #R0278) supplemented with the addition of protease and phosphatase inhibitors (Beyotime, CAT #P1051). After brief sonication, the mixture was pelleted at 12,000 *g* for 10 min at 4 °C and the protein supernatant was transferred to another tube. Proteins from whole-cell lysates were resolved by 10% sodium dodecyl sulfate-polyacrylamide gel electrophoresis and then transferred to PVDF membranes. Anti-NOX4 (Novus, CAT #NB110-58851), anti-β-actin (Novus, CAT #NB600-501), anti-p-SMAD3 (Abcam, #ab52903), anti-SMAD3 (Abcam, #ab40854), anti-p-ERK (CST, CAT #4370), anti-ERK(CST, CAT #4695), anti-p-AKT (CST, CAT #4060), and anti-AKT (CST, CAT#9272) antibodies were used at 1:1000 and secondary antibodies (Beyotime, CAT #A0208) conjugated with horseradish peroxidase (HRP) were used at 1:1000 dilutions in 5% non-fat dry milk. After the final washing, PVDF membranes were exposed for an enhanced chemiluminescence assay using the Gel imaging instrument (Shang Hai CLiNX, China). Quantification of western blot was performed using Image J software.

### ECAR

Extracellular acidification rate (ECAR) was measured using a Seahorse XF24 analyzer (Seahorse Bioscience). Briefly, ECAR was determined using the Glycolysis Stress kit (Seahorse Biosciences) according to the manufacturer’s standard protocol. All assays were performed with *N* = 3 or more per condition and repeated three times. Injections were performed as follows: glucose (20 mM), oligomycin (4.5 μM), 2-DG (100 mM), GOD (100 μg/mL, MERCK, CAT #G7141), NAC (10 mM, MERCK, CAT #A7250). Results were analyzed using Seahorse Wave version 2.4. The experiment was replicated three times.

### Database

Correlation between the transcriptive expression levels of LDHA, HIF1α, BCL2, Glut1, Ki67, and NOX4 was analyzed using data from TCGA by GEPIA (http://gepia.cancer-pku.cn/).

### Statistical analysis

Student’s *t* test (two-tailed, unpaired) was used for two-group comparisons. For comparisons of three or more groups, one-way ANOVA or two-way ANOVA was performed. The results from three independent experiments are presented as mean ± SD. All statistical analyses were performed using the GraphPad Prism software and means were compared. The Kaplan-Meier log-rank test was used for survival comparison. The sample size for all experiments was not chosen with consideration of adequate power to detect a pre-specified effect size. All statistical tests are justified as appropriate, and data meet the assumptions of the tests. There is an estimate of variation within each group of data. The variance is similar between the groups that are being statistically compared. Asterisks denote statistical significance as follows: NS, no significance; *P* > 0.05; *P ≤ 0.05; **P ≤ 0.01; ***P ≤ 0.001.

## Supplementary information

Author Agreement Form

Original Data File

<i>Supplementary Figures</i>

## Data Availability

All data needed to evaluate the conclusions in the paper are present in the paper and/or the Supplementary Materials.
