# Supplementary figures and images for: Targeting NOX4 disrupts the resistance of papillary thyroid carcinoma to chemotherapeutic drugs and lenvatinib
Source: Cell Death Discov. 2022 Apr 8;8:177. doi: 10.1038/s41420-022-00994-7 (PMC8990679; doi:10.1038/s41420-022-00994-7)

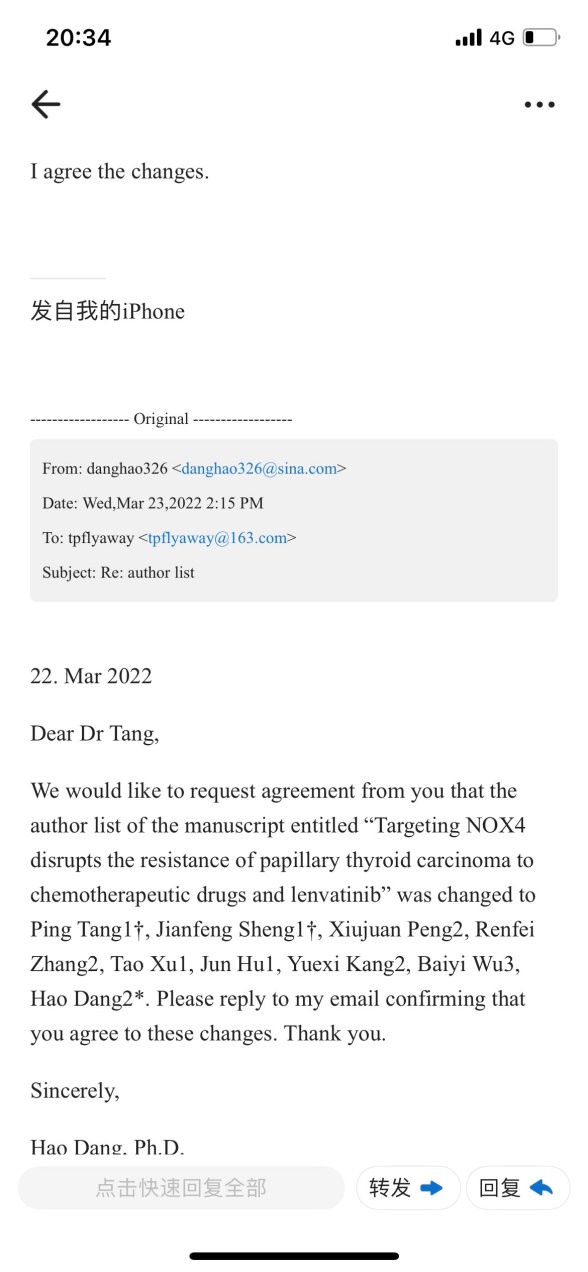

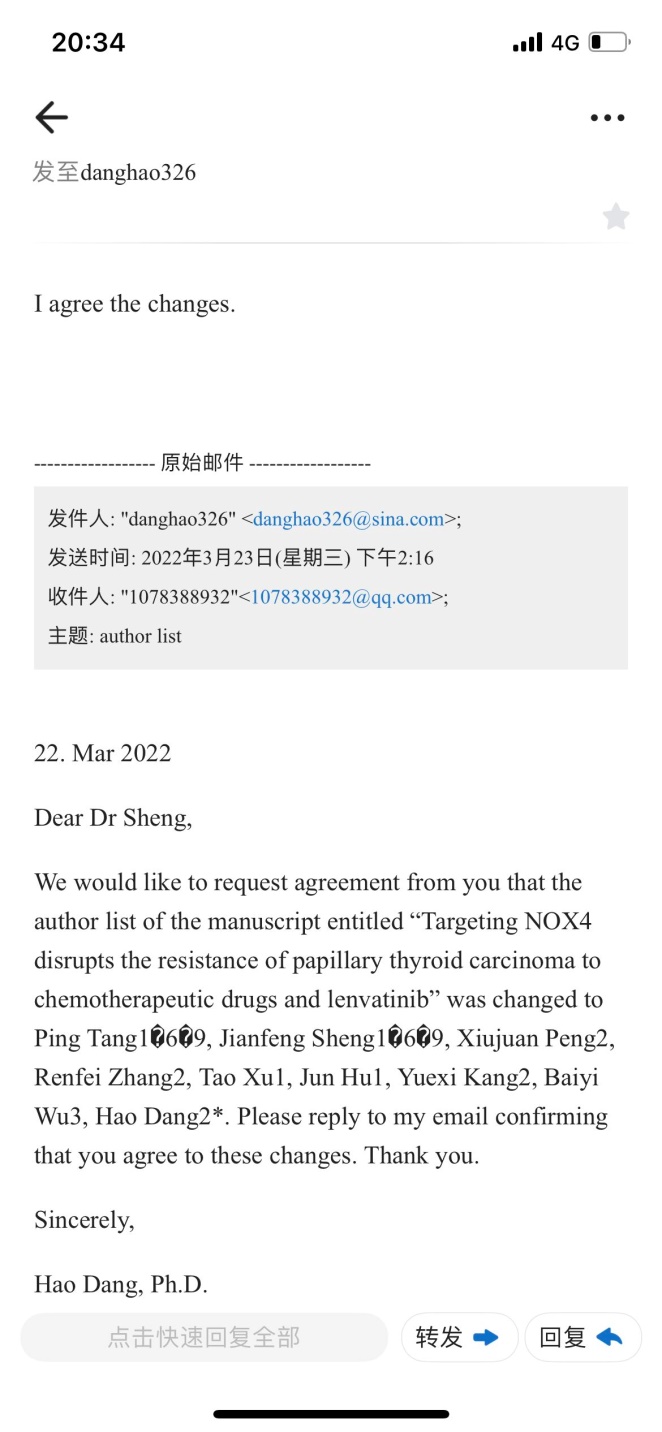


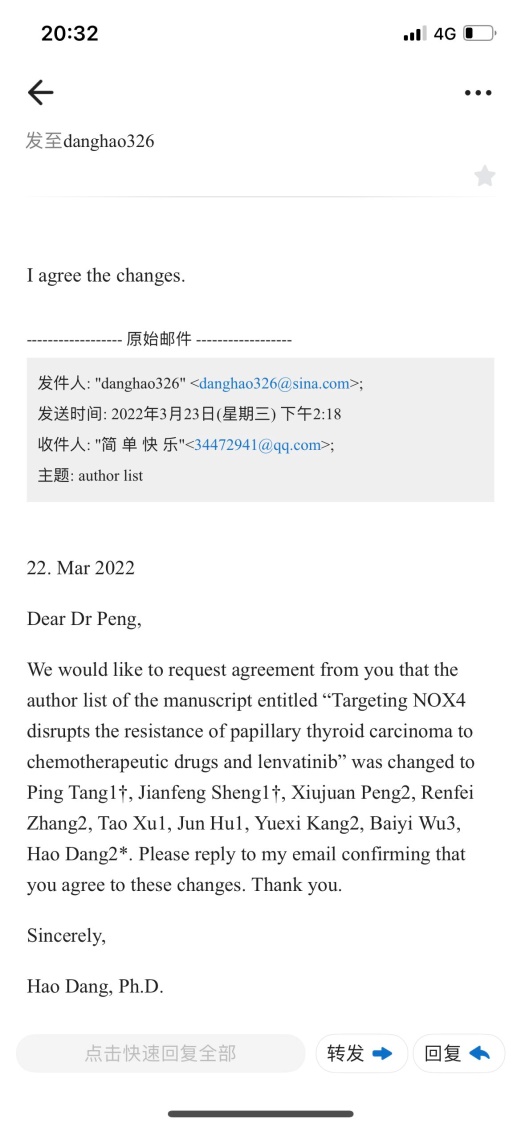

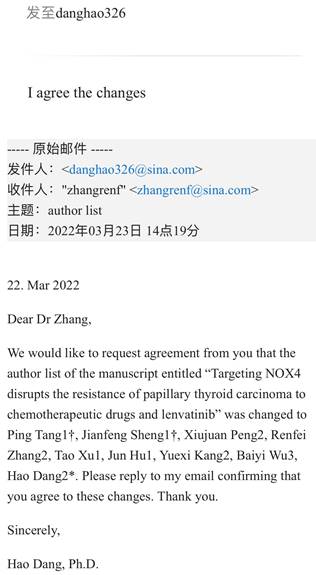


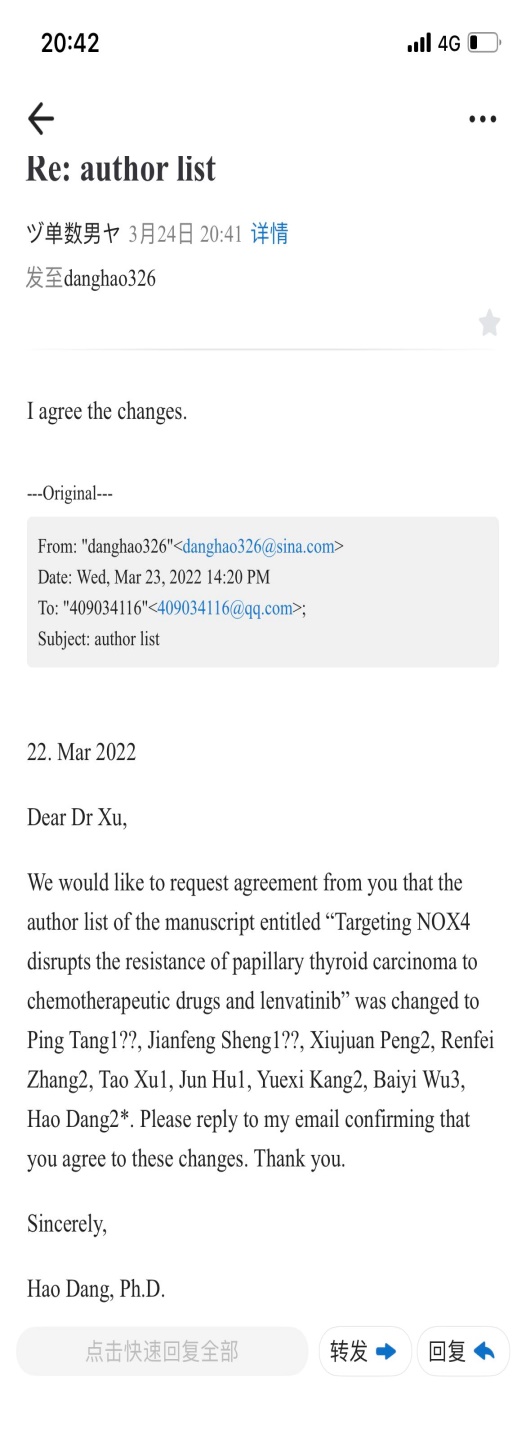

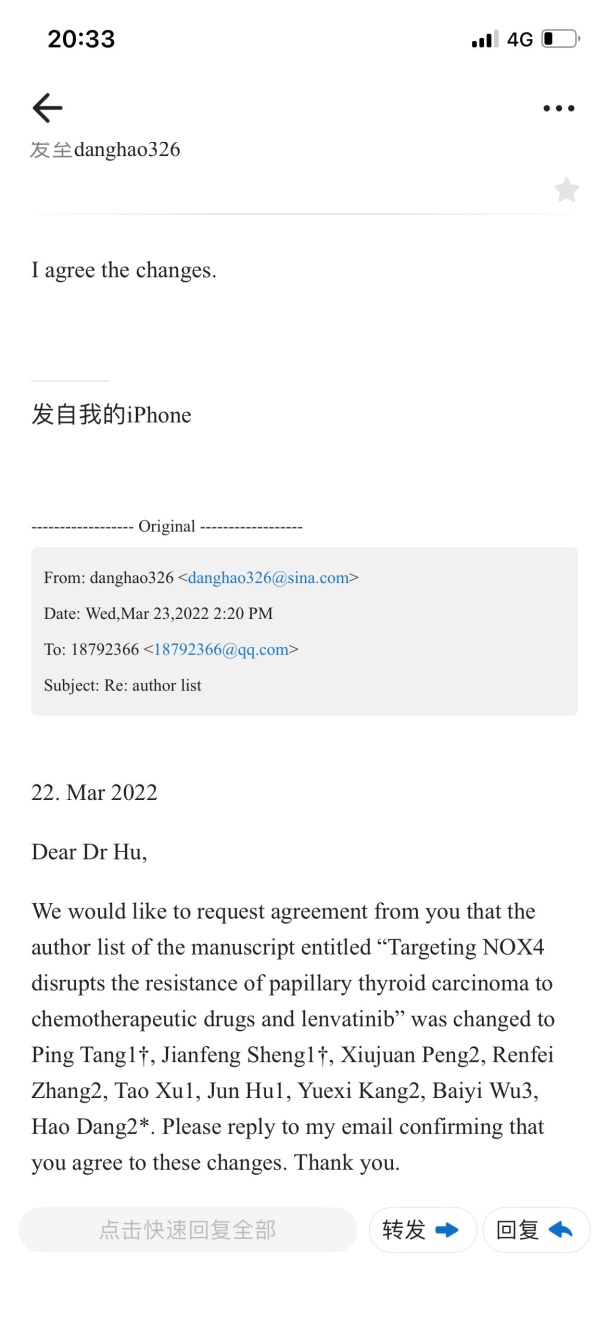


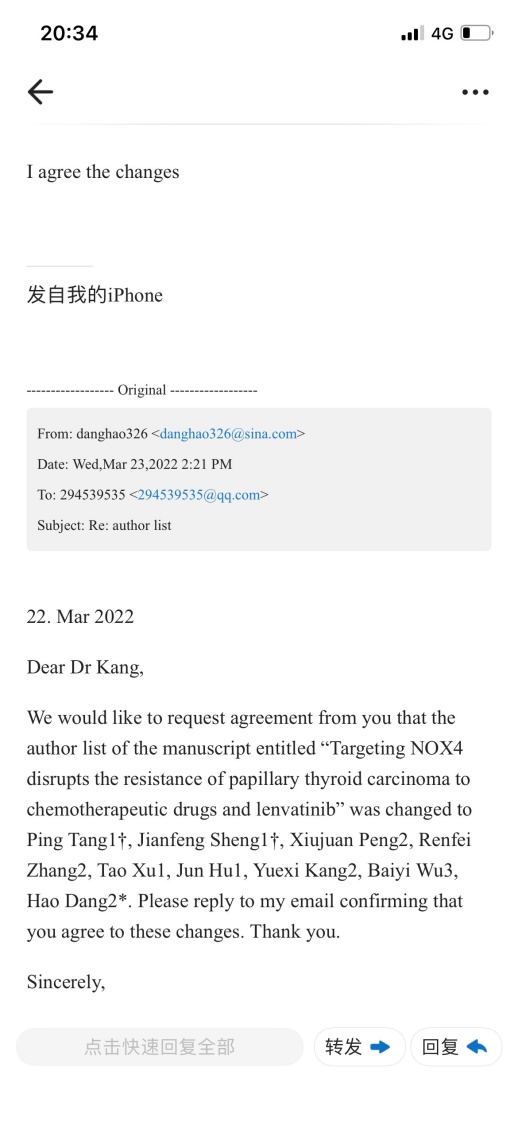

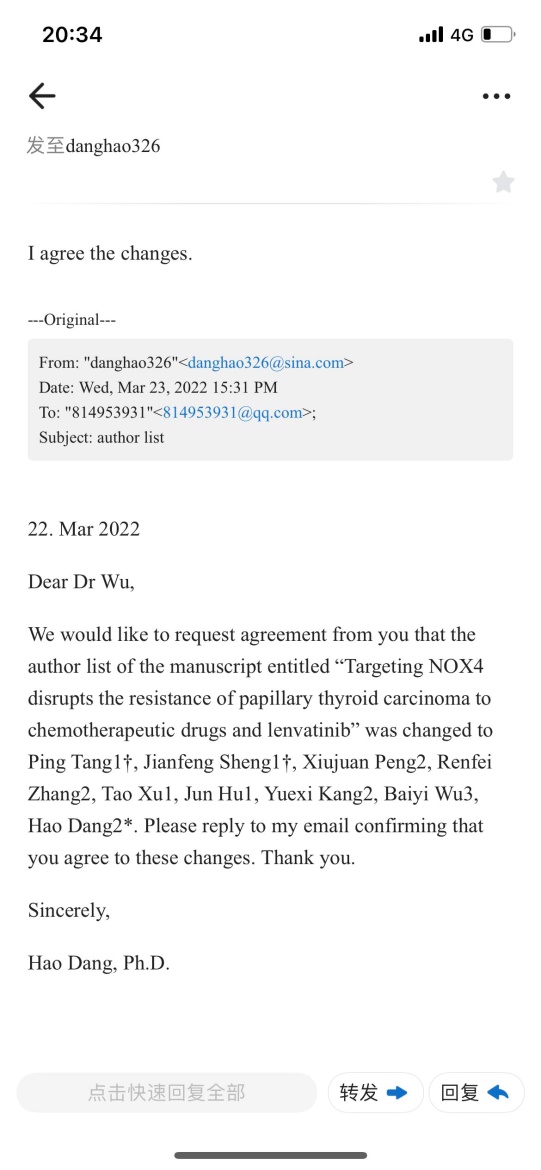

Supplement: Supplementary file 1 — Author Agreement Form [file 41420_2022_994_MOESM1_ESM.docx]
