# Supplementary material for: Targeting NOX4 disrupts the resistance of papillary thyroid carcinoma to chemotherapeutic drugs and lenvatinib
Source: Cell Death Discov. 2022 Apr 8;8:177. doi: 10.1038/s41420-022-00994-7 (PMC8990679; doi:10.1038/s41420-022-00994-7)

Original images for Blots, Related to Figure 1A

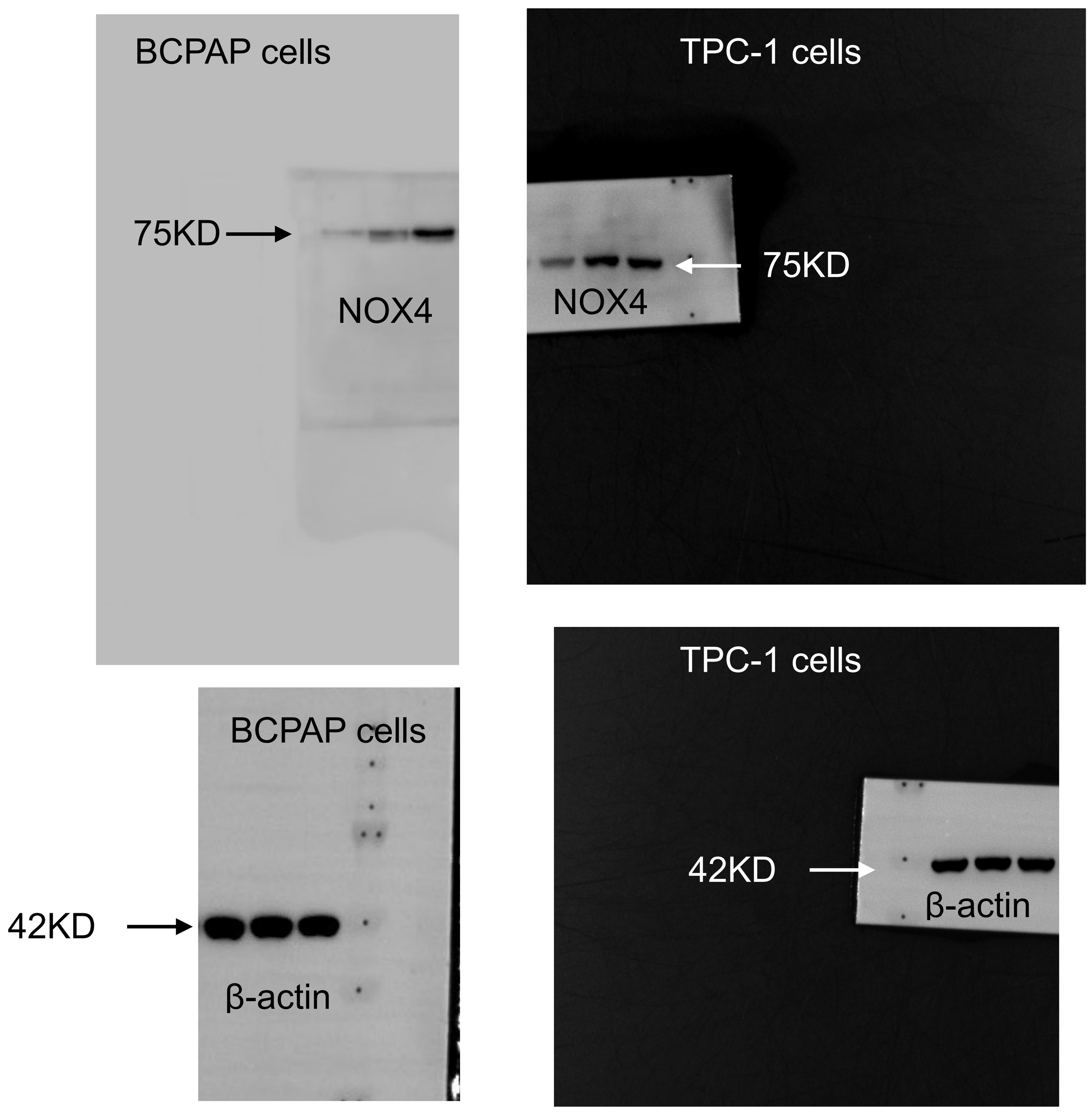

Original images for Blots, Related to Figure 6A

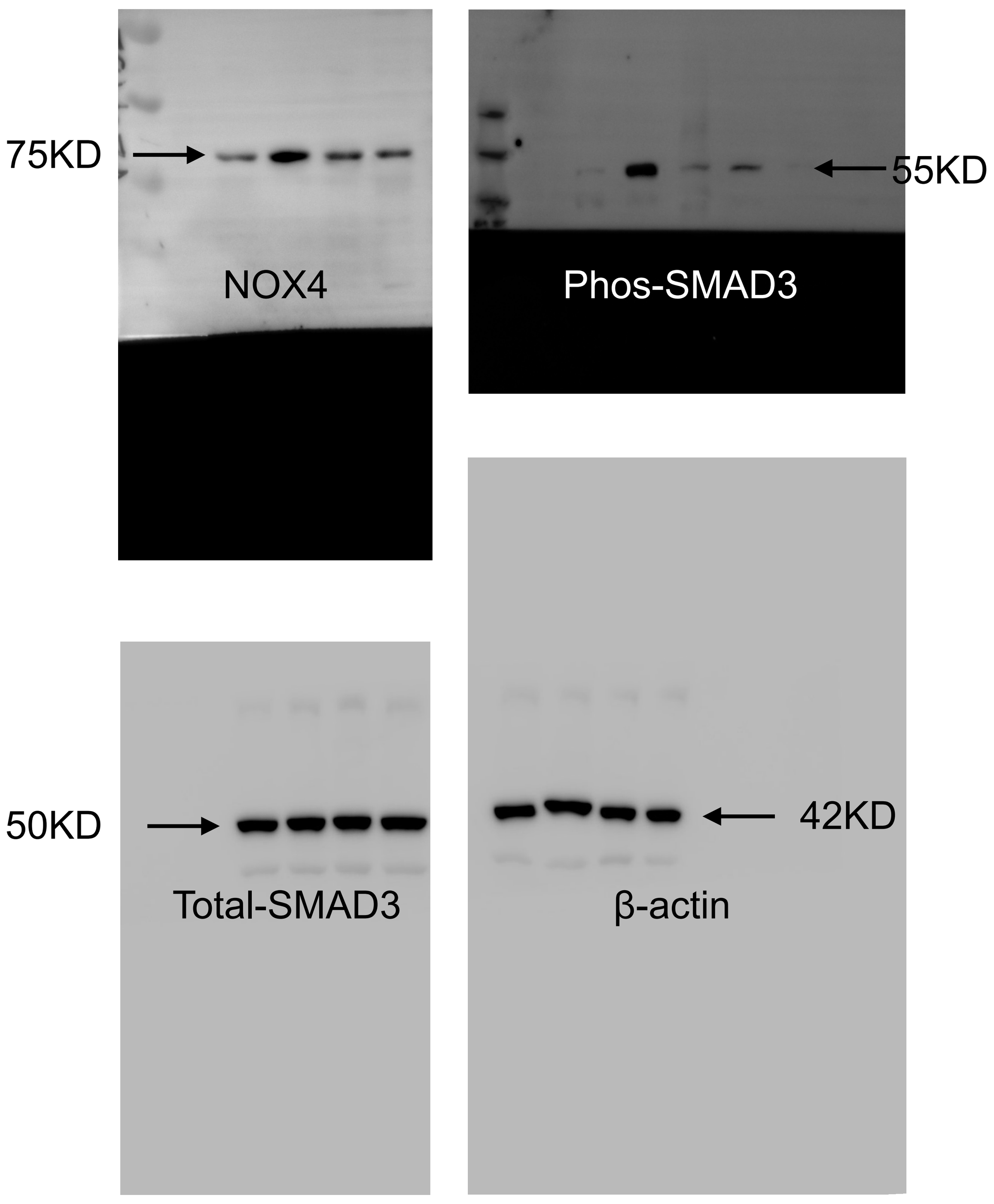

Original images for Blots, Related to Figure 6B

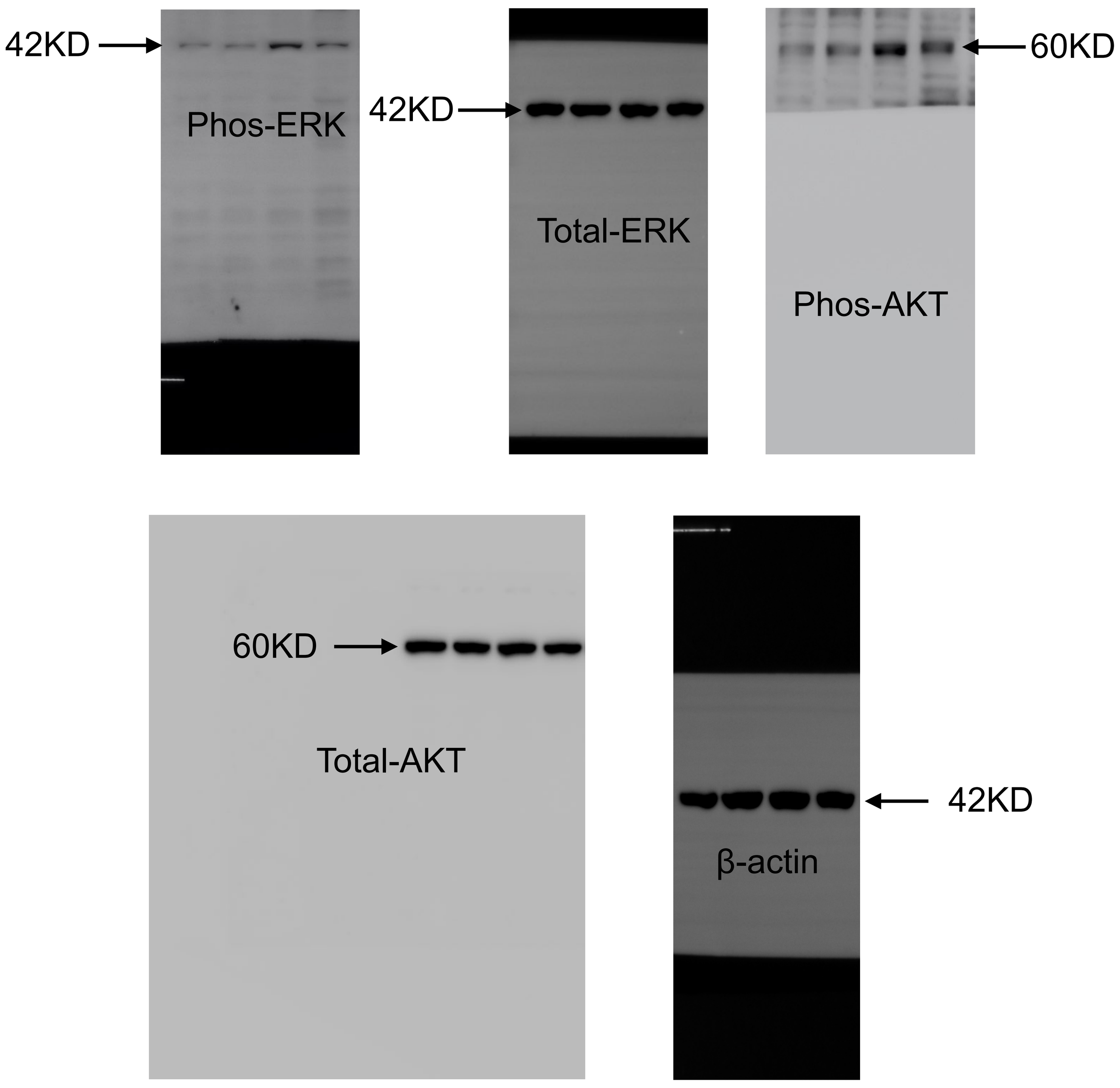

Original images for Blots, Related to Figure 6F

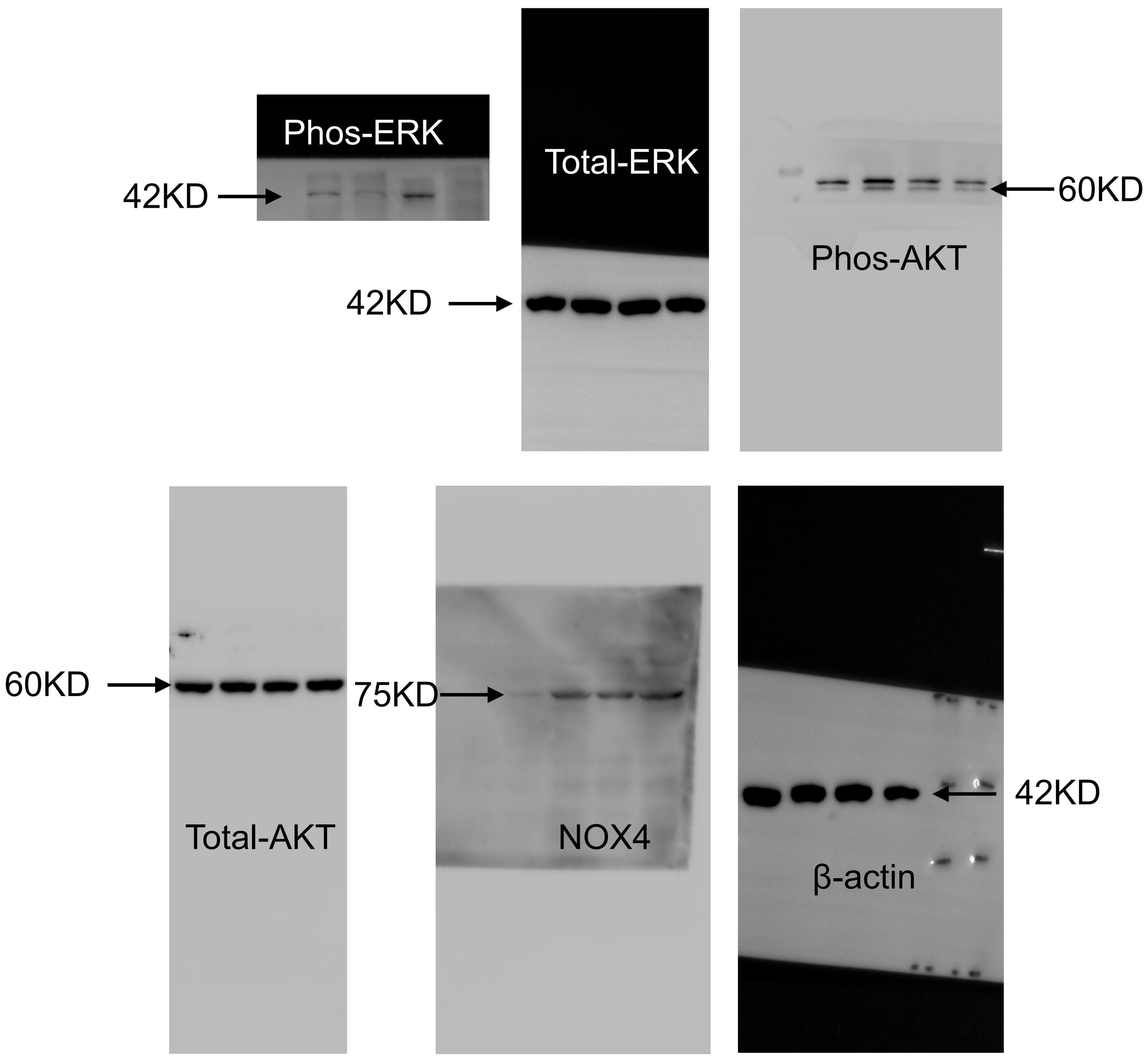

Original images for Blots, Related to Supplementary Figure 1A

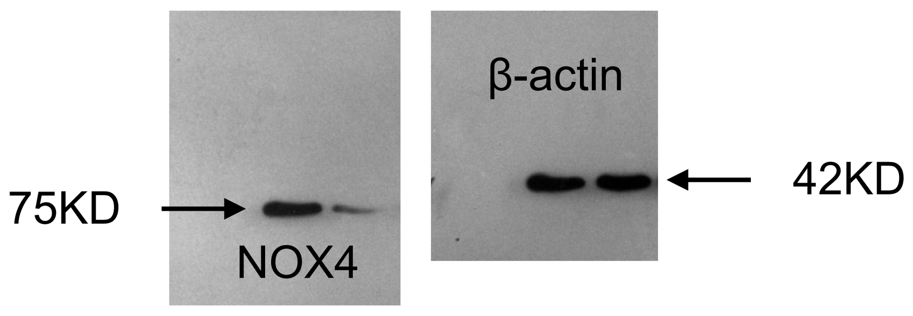

Original images for Blots, Related to Supplementary Figure 3B

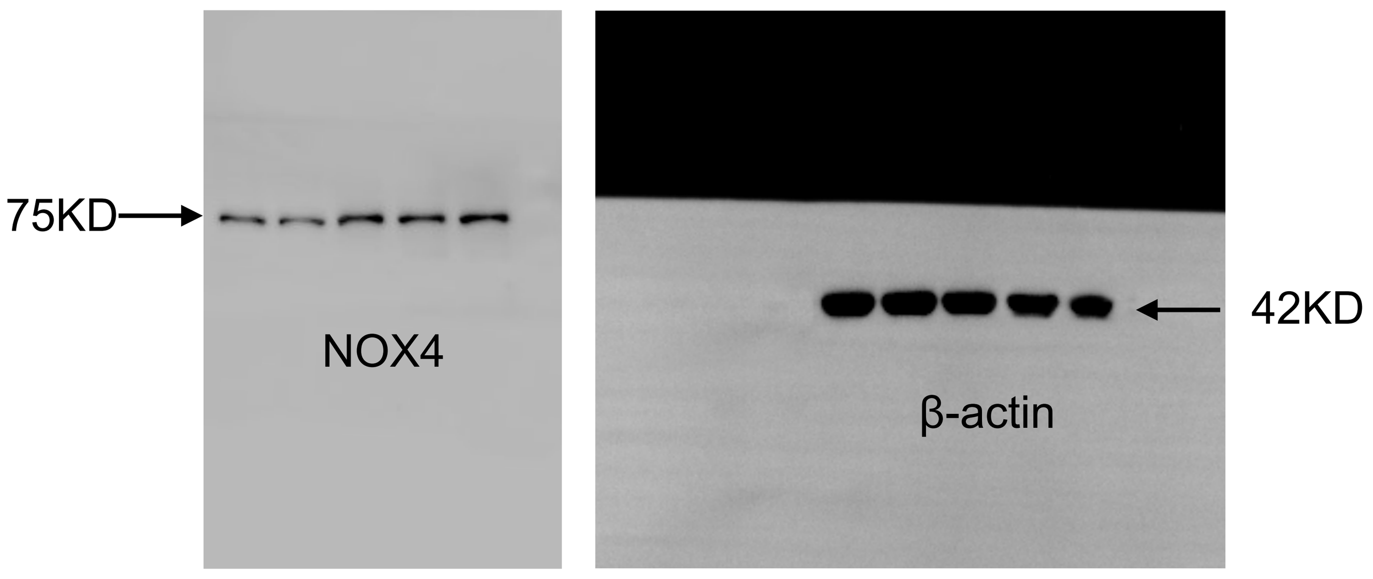

Original images for Blots, Related to Supplementary Figure 4B

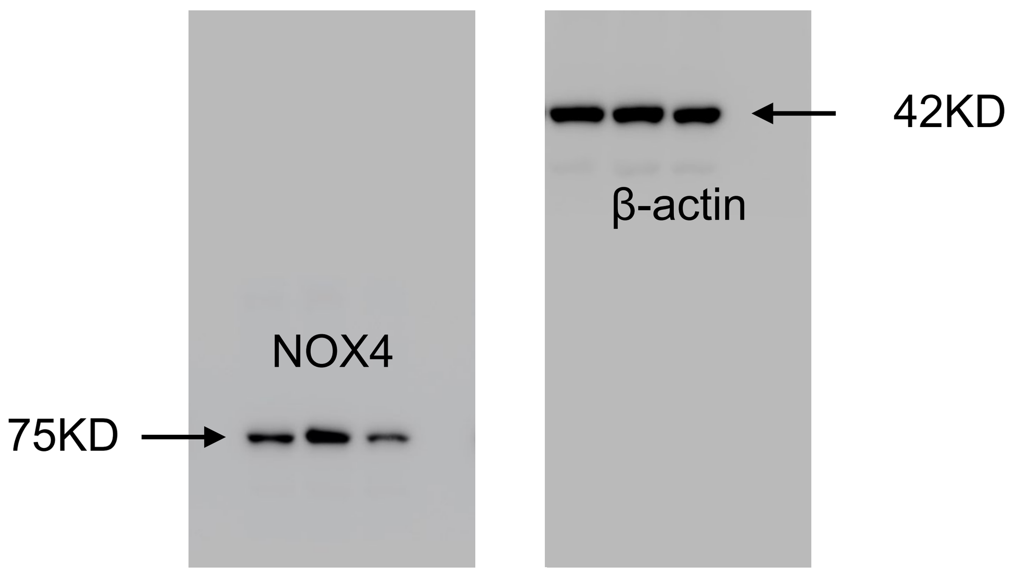

Supplement: Supplementary file 2 — Original Data File [file 41420_2022_994_MOESM2_ESM.pdf]
