# Supplementary material for: Targeting NOX4 disrupts the resistance of papillary thyroid carcinoma to chemotherapeutic drugs and lenvatinib
Source: Cell Death Discov. 2022 Apr 8;8:177. doi: 10.1038/s41420-022-00994-7 (PMC8990679; doi:10.1038/s41420-022-00994-7)
Supplement: Supplementary file 3 — Supplementary Figures [file 41420_2022_994_MOESM3_ESM.docx]

**Targeting NOX4 disrupts the resistance of papillary thyroid carcinoma to chemotherapeutic drugs and lenvatinib**

Ping Tang, Jianfeng Sheng, Xiujuan Peng, Renfei Zhang, Tao Xu, Jun Hu, Yuexi Kang Baiyi Wu, Hao Dang

*Supplementary Figures*


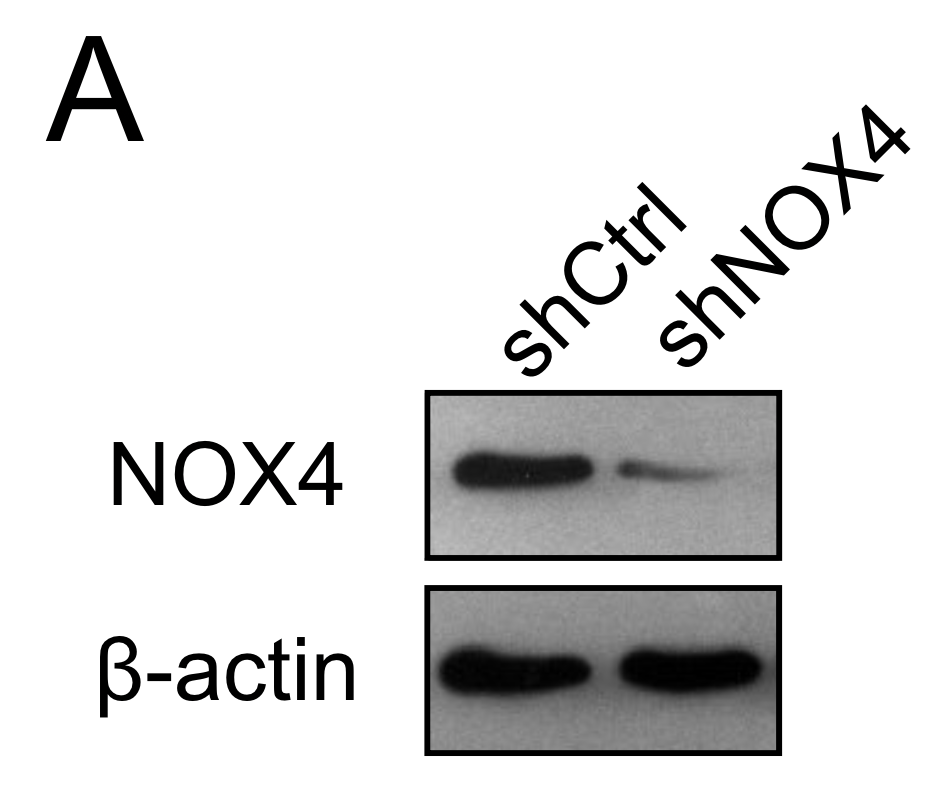


**Figure S1. NOX4 is under-regulated in BCPAP cells expressing shNOX4. Related to Figure 1.** (A) Immunoblots of BCPAP cells expressing control shRNA and shRNA against NOX4.


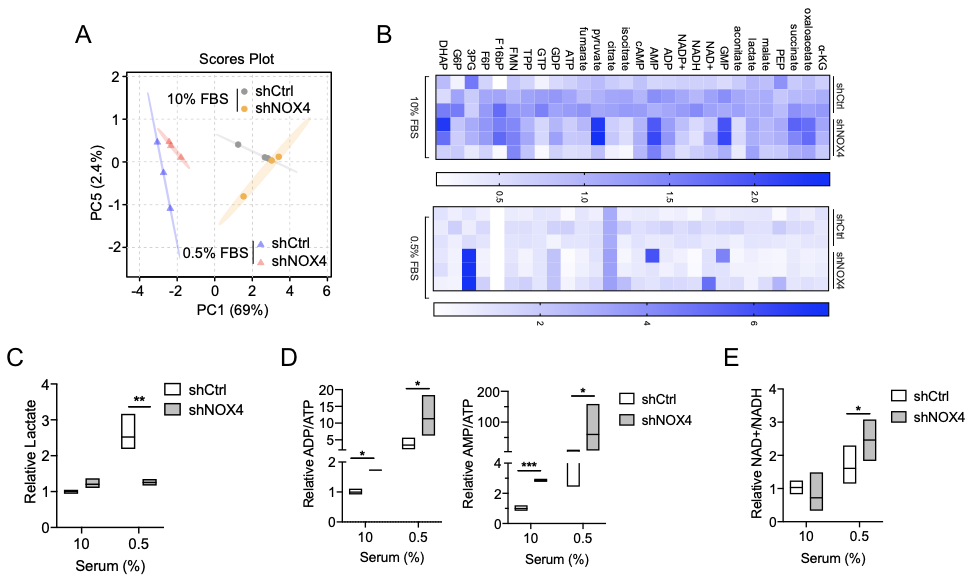


**Figure S2. NOX4 deficiency modulates the levels of energy-associated metabolites in BCPAP cells.** (A) The PCA showing the alteration due to either shRNA against NOX4 or serum starvation for 48 h in BCPAP cells. (B) Heat-maps of energetic metabolites in BCPAP cells expressing shRNA against NOX4 or not 48 h post treatment with 10% FBS or 0.5% FBS (n=3, Biological Repeats). (C-E) Relative levels of the indicated metabolites or ratio based on data from targeted metabolomics. All values were normalized by the mean value of shCtrl under 10% FBS. Box plots show the mean and the upper and lower 25%, respectively. **P*<0.05, ***P*<0.01, ****P*<0.001.


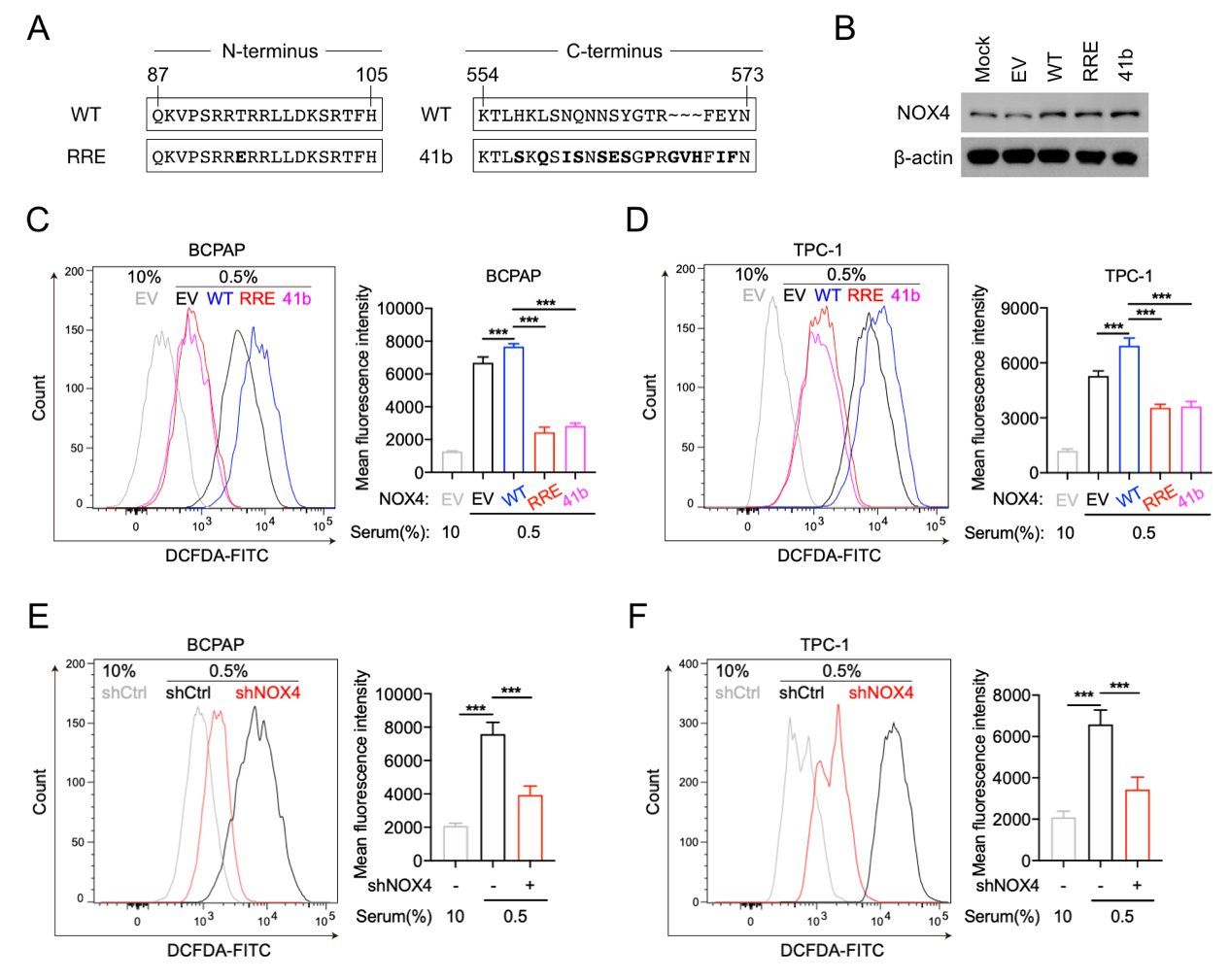


**Figure S3. The positive effect of NOX4 in starved PTC cells. Related to Figure 2.** (A) Sequence alignment of NOX4^RRE^ and NOX4^41b^. Numbers refer to the amino acid position in NOX4 WT. (B) Expressions of NOX4 protein were analyzed by immunoblotting. Mock, un-transfected BCPAP cells. EV, empty vector. WT, wild type. (C-F) Flow cytometry (left) and the statistical analyses (right) showing cellular ROS in cells with indicated treatment. WT, Wild type. RRE and 41b, two mutated forms of human NOX4. ****P*<0.001.


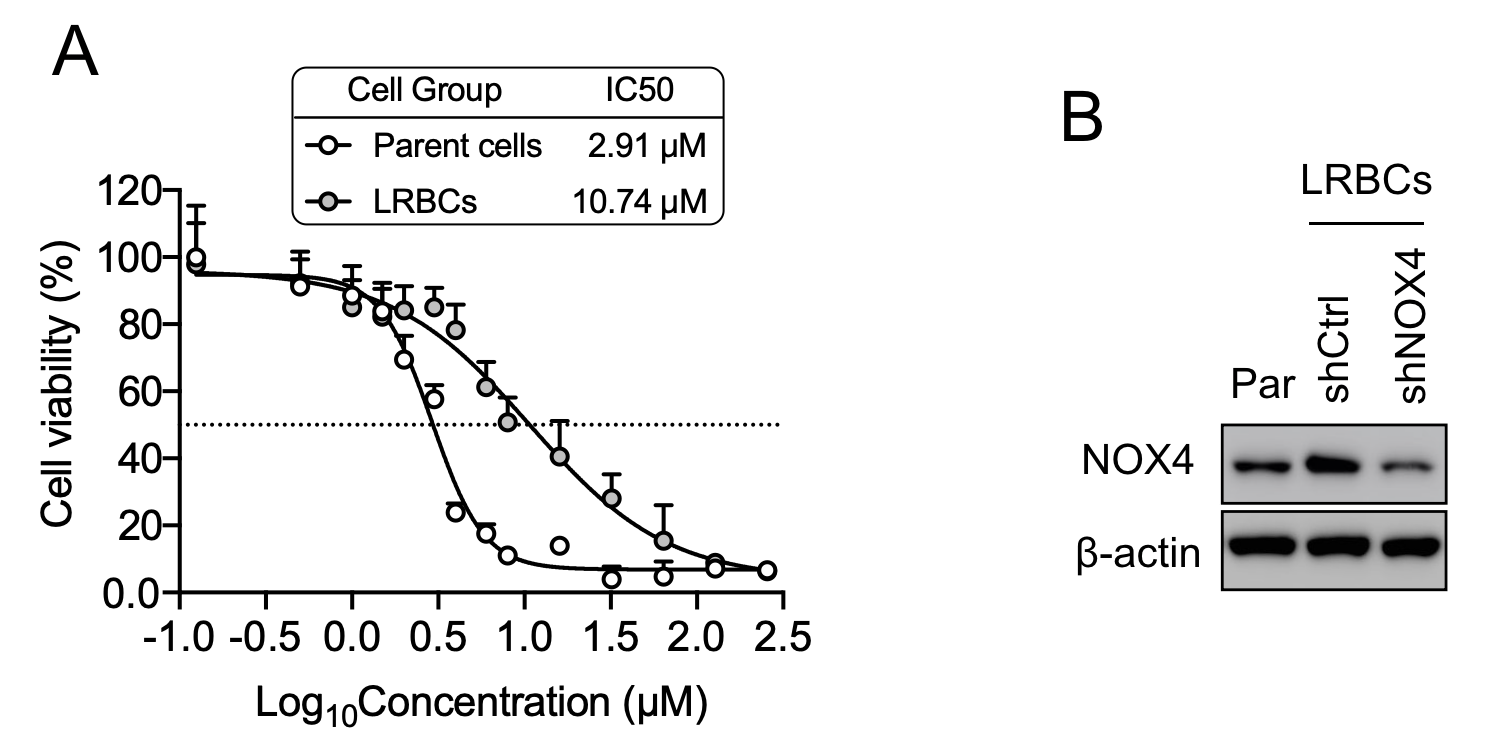


**Figure S4. IC50 calculation of LRBCs and immunoblots for LRBCs expressing shCtrl and shNOX4. Related to Figure 4.** (A) IC50 curves exhibit precise inhibition of lenvatinib in the parental cells and LRBCs. (B) Immunoblots showing NOX4 protein expression in the parental cells, LRBCs expressing shCtrl and LRBCs expressing shNOX4.


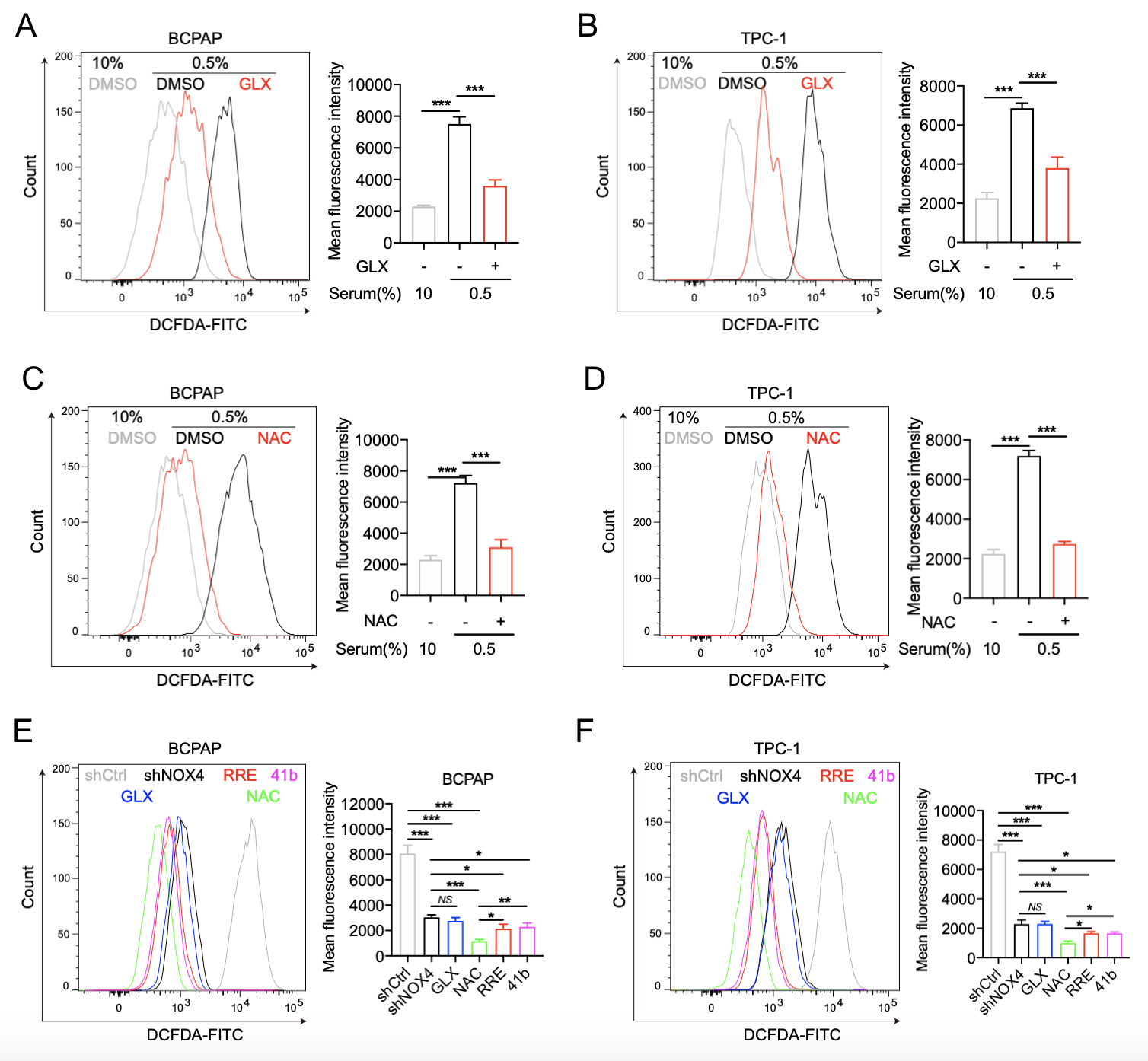


**Figure S5. The impact of inhibiting NOX4-derrived ROS by inhibitors or dominant-negative mutants on ROS. Related to Figure 5.** (A-F) Flow cytometry (left) and the statistical analyses (right) showing cellular ROS in the cells treated with the indicated way. WT, Wild type. RRE and 41b, two mutated forms of human NOX4. **P*<0.05. ***P*<0.01. ****P*<0.001. *NS*, no significance.


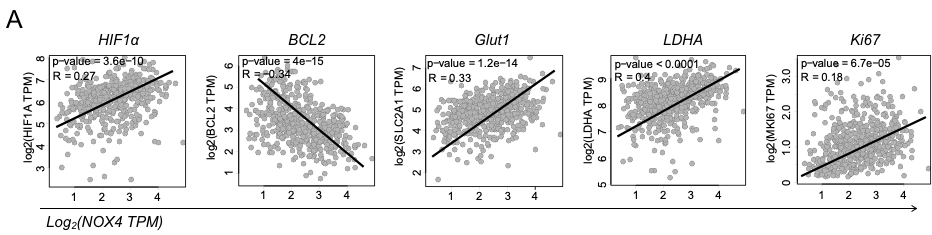


**Figure S6. NOX4 correlates with five markers in tumor of patients with thyroid cancer. Related to Figure 7.** (A) Correlation between NOX4 expression and HIF1a, BCL2, Glut1, LDHA and Ki67 expression in tumor of patients with thyroid cancer.
